# The Relationship Between Motivation for Physical Activity, Physical Activity Level, and Body Mass Index for University Students

**DOI:** 10.3390/sports13040096

**Published:** 2025-03-25

**Authors:** Stefan Alecu, Gheorghe Adrian Onea, Dana Badau

**Affiliations:** Faculty of Physical Education and Mountain Sports, Transilvania University, 500068 Brasov, Romania; alecu.stefan@unitbv.ro (S.A.); dana.badau@unitbv.ro (D.B.)

**Keywords:** physical activity, intrinsic and external motivation, BMI, health behavior, proactive behavior

## Abstract

The object of the study was to identify the relationship between the motivation for physical activity, the physical activity index (PAI), and the body mass index (BMI) for a sample of university students, taking into consideration the gender difference. The study subjects were 813 (340 men and 473 women), and the age range of the participants varied between 18 and 25 years. The RM 4-FM (Motivation for Physical Activity and Exercise) and PAI online questionnaires were applied to measure the motivation and the physical activity level. The study’s novelty focused on analyzing how intrinsic and extrinsic motivation interact and correlate BMI and physical activity levels (PAI). Regarding gender, we approached the identification of motivational differences and the level of physical activity of students from non-sports faculties to identify relevant aspects for promoting proactive behaviors. Results revealed significant gender differences: men had a higher PAI (53.48 ± 28.11) than women (36.67 ± 23.92, *p* < 0.01); BMI (23.25 vs. 21.69, *p* < 0.01). The Cohen’s value of RAI of RM 4-FM, BMI, and PAI were > 0.5, indicating a large effect size. The analysis presents a unique perspective of the interactions between psychological factors, motivation, and physical characteristics, providing insight into developing strategies to improve and promote a proactive lifestyle.

## 1. Introduction

Physical activity is known to be crucial for both physical and mental health and is associated with the prevention of chronic disease, improvement of well-being, and a healthy lifestyle [1,2]. However, the level of physical activity of the young population has decreased significantly in recent decades, causing concern for public health [3,4]. Motivation plays a fundamental part in starting and maintaining an active behavior and is directly connected to the level of involvement and persistence in physical activities [5,6,7]. The experts underline the importance of intrinsic and extrinsic motivation and highlight that inner motivation, grounded in pleasure and personal satisfaction, is the most sustainable form [8,9,10]. Motivation is a critical factor in initiating and maintaining physical activity, which can be classified into two distinct categories: intrinsic and extrinsic motivation. Intrinsic motivation is characterized by participation in physical activity for the sake of enjoyment and personal fulfillment. Conversely, extrinsic motivation is influenced by external rewards or social pressures. According to self-determination theory (SDT), extrinsic motivation can be further segmented into various forms, including external regulation, which is motivated by rewards or social approval, and introjected regulation, which is driven by feelings of guilt or obligation [11]. Understanding these distinctions is vital for developing targeted strategies aimed at enhancing engagement in physical active engagement. Psychological and motivational factors are codependent on individual physical characteristics, such as body mass index (BMI), an important indicator of health status [12,13]. The precedent studies showed significant differences between men and women regarding the levels of physical activity and motivation, which are influenced by social, cultural, and biological factors [14]. The correlations between motivation, the level of physical activity, and BMI provide an essential and innovative perspective for developing a personalized approach to promoting proactive behavior [15,16]. Despite the extensive research conducted on the complex relationship between motivation and physical activity, a majority of studies fail to address the synergistic effects among motivation, body mass index (BMI), and physical activity levels (PAI), especially within the dynamic context of a university student population [17]. While gender differences in motivation have been acknowledged in several studies, there exists a significant gap in the literature regarding the relationship between various forms of motivation—specifically intrinsic and extrinsic motivation—and their correlation to BMI and PAI in young adults [18,19]. This study aims to elucidate the fundamental factors that influence physical activity behaviors among university students, thereby enhancing the understanding of how motivation interacts with health metrics in this distinctive demographic.

Analyzing the motivation for physical activity, the physical activity index (PAI), and BMI, also considering gender differences and the living environment, is a complex, innovative approach. Among the objectives, the following can be mentioned: evaluation of the correlations between motivation and physical parameters, as well as the identification of the most effective strategies to promote an active lifestyle among the youth population [20,21,22,23].

The research contributes significantly to the understanding of the factors that influence the choice of proactive behavior. The motivation for physical activity is a subject thoroughly studied by sports and public health sciences, and it is considered an essential factor for the promotion of an active and healthy lifestyle. Countless studies stressed the influence of intrinsic and extrinsic motivation over involvement in physical activities, highlighting the importance of long-term motivation to obtain real benefits [24,25,26]. Studies have shown that motivation analysis can be approached from the perspective of the role of autonomy, competence, and social relationships as support for engaging in physical activities [27,28,29].

The physical activity index (PAI) and the body mass index (BMI) are among the indicators frequently used to assess the relationship between the level of physical activity and health. The research suggests that higher levels of physical activity are associated with lower values of BMI and a decrease in risks for health, such as cardiovascular diseases or obesity [30,31]. Intrinsic motivation, characterized by satisfaction and pleasure of movement, is considered a significant predictor of long-term involvement in physical activities [32,33,34]. Moreover, the frequently analyzed gender differences revealed that men tend to obtain higher scores for PAI than women, as the latter have a stronger correlation between intrinsic motivation and their general physical health [35,36]. Also, the sociocultural factors and the individual and genre understanding of physical activity play an important part.

Based on the previous arguments, the present research can bring new evidence to the understanding of the complex relationships between motivation, the level of physical activity, and BMI in a specific approach for a big and diverse sample of university students. The aim of the study was to identify the relationship between the motivation for physical activity, the physical activity index (PAI), and the body mass index (BMI) for a sample of university students, taking into consideration the gender difference. Physical activity is vital for health, but it forms only one aspect of a broader lifestyle framework impacting university students’ well-being. Studies show that university life frequently correlates with sedentary habits, inconsistent eating patterns, elevated stress, and sociocultural factors, all of which influence health results [31,37,38,39].

The proactive and health specific objectives of the study:

O1. A detailed exploration of how intrinsic and extrinsic motivation influences the levels of physical activity and health of the male and female groups;
O2. The combination of the assessment of the motivational parameters (RM 4-FM), BMI, and PAI in an integrated perspective;
O3. As students are a vulnerable group to the health risks associated with a sedentary lifestyle, the research provides relevant information for the motivation for physical activity corroborated to BMI and PAI.

## 2. Materials and Methods

### 2.1. Design of the Research

The period of research spanned between October and November 2024. During this time, the data about the characteristics of the subjects of the study (age, gender, weight, height, residence) and the answers to the two questionnaires RM 4-FM: Motivation for Physical Activity and Exercise (in two parts) and the questionnaire for the physical activity index (PAI) were collected online, via Google Forms. The RM 4-FM scale (Motivation for Physical Activity and Exercise) was specifically selected for this study due to its robust and comprehensive evaluation of intrinsic and extrinsic motivation. Its well-structured subscales resonate strongly with self-determination theory (SDT), making it an ideal choice for our research. This instrument has been extensively validated in previous studies involving university student populations, demonstrating its effectiveness [40,41]. Furthermore, it offers an in-depth analysis of motivational factors, including external regulation, introjected regulation, identified regulation, and intrinsic motivation, ensuring a thorough understanding of what drives physical activity among students. The indicator for the area of residence was coded with value 1 for rural areas and 2 for urban areas. The questionnaires were created in Google Forms, and the links were distributed on the web to the student groups. The principles of the Declaration of Helsinki were respected in this study. All the subjects participated voluntarily. The study was approved by the Ethical Board of the Faculty of Physical Education and Mountain Sports of Transylvania University of Brasov, approval no. 246/01.10.2024.

### 2.2. Participants

The number of participants included in the study was 813 students in the first and second years of study at Transylvania University. The students included in this study are enrolled in all 17 faculties of the university (excluding students at the Faculty of Physical Education and Mountain Sports). In addition to independent physical activities, the study subjects have a compulsory physical education discipline included in the academic curriculum. The main activities carried out under the coordination of an expert with a PhD were fitness, badminton, basketball, volleyball, football, general physical development, and other disciplines. Participants were recruited using a convenience sampling method. Students voluntarily filled out questionnaires distributed through Transilvania University’s communication channels. The students involved in the study come from various academic programs to ensure a diverse representation across disciplines. The subjects formed two groups: the male group, with 340 (41.9%) subjects, and the female group, with 473 (58.1%) subjects. The eligibility criteria include active students aged between 18 and 25 years, good health status, and thorough responses to the questions. Initially, 838 students completed the questionnaires, but 25 were ruled out due to incomplete data. The characteristics of the study groups (age, BMI, residence) are shown in Table 1.

**Figure 1:**
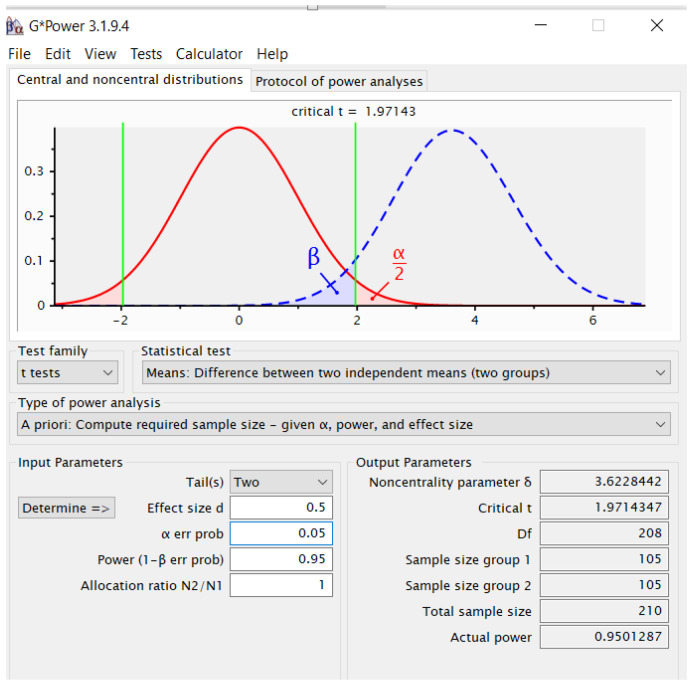
GPower analysis of sample size.

We used GPower 3.1.94 for the study (Figure 1), keeping a medium effect size in mind. We selected a two-sided test with a significance level set at α = 0.05 and a power level of 0.8 (corresponding to β = 0.2). Additionally, we decided to maintain equal sample sizes for both groups. For the calculations, we chose “two” from the “tail(s)” drop-down menu. We entered the following values: an effect size (d) of 0.5, a significance level (α) of 0.05, a desired power (1 − β) of 0.95, and an allocation ratio (N2/N1) of 1. After pressing the “calculate” button, the results indicated that each group would require 105 participants, leading to a total sample size of 210, as displayed in the output parameters.

### 2.3. Measurements

The body mass index (BMI) is an indicator of weight, calculated by the formula:

BMI = weight (kg)/[height (m)]^2^

The normative values for men and women aged between 18 and 25 are as follows: under 18.5—underweight (mild thinness); 18.5–24.9—normal range; 25–29.9—overweight (pre-obese), and over 30—obese (class I).

#### RM 4-FM: Motivation for Physical Activity and Exercise [42]

RM 4-FM is the instrument used to analyze the motivation for physical activity and exercise, divided into two parts: Motivation for Physical Activity (16 items) and Motivation for Workout (12 items). The assessment scale: the subjects grade the motifs on a scale from 1 (completely disagree) to 7 (completely agree). The relative autonomy index (RAI) uses negative values to indicate extrinsic motivation, influenced by external factors (social pressure, rewards); the positive values indicate extrinsic motivation, grounded on inner satisfaction and values.

Part 1. RM 4-FM—Motivation for Physical Activity, subscales, and corresponding items:

- External Regulation: Motivation is guided by external factors, such as rewards, social approval, or pressure exercised by peers; associated items: 2, 7, 11, 14.
- Introjected Regulation: Reflects internal pressure, such as guilt or the desire to avoid disapproval; associated items: 1, 4, 6, 13.
- Identified Regulation: Motivation is associated with the recognition of the personal value of the physical activity; associated items: 5, 9, 12, 16.
- Intrinsic Motivation: Physical activity is conducted for self-satisfaction and well-being; associated items: 3, 8, 10, 15.

Part 2. RM 4-FM: Motivation for Exercise/Workout, subscales, and corresponding items:

- External Regulation: Refers to external factors of motivation (desire to win other’s approval or fear of criticism); associated items: 5, 7, 12.
- Introjected Regulation: Highlights internal pressures (guilt or fear of criticism); associated items: 3, 6, 9.
- Identified Regulation: Based on the recognition of the value of physical activity (health or personal objectives); associated items: 2, 8, 10.
- Intrinsic Motivation: Physical activity for well-being or personal satisfaction, considered the most sustainable form of motivation; associated items: 1, 4, 11.

The physical activity index (PAI) [43] is a method of evaluating the level of physical activity based on the intensity, duration, and frequency of the activity. The calculation formula of PAI is:

PAI = intensity × duration × frequency

The parameters and associated scores for intensity are as follows: 5 points—intense activity, leading to accelerated breathing (panting) and abundant sweat; 4 points—intermittent accelerated breathing and sweat (e.g., tennis game); 3 points—significant effort, proper to recreational sports (e.g., cycling); 3 points—moderate effort, specific to recreational sports (e.g., volleyball); 1 point—low effort (walking, fishing). The parameters and associated scores for duration are as follows: 4 points for over 30 min, 3 points for between 20 and 30 min, 2 points for between 10 and 20 min, and 1 point for under 10 min. The parameters and associated scores for frequency are as follows: 5 points for daily or almost daily activity (maximum one day of pause per week); 4 points for 3 to 5 sessions per week (maximum two days between sessions); 3 points for 1–2 weekly sessions (on non-consecutive days); 2 points for a few sessions per month; 1 point for less than once per month. PAI score interpretation: 80–100 points—very active lifestyle (high level); 60–80 points—active lifestyle (very good); 40–60 points—moderately active lifestyle (reasonable); 20–40 points—slightly active or insufficiently active lifestyle (weak); under 20 points—sedentary lifestyle (very weak).

### 2.4. Statistical Analysis

The software IBM-SPSS 30 was used for the statistical analysis of the data, allowing for a rigorous and detailed assessment of the parameters included in the research: arithmetic mean (X) and standard deviation (SD). The mean difference between tests (∆X), associated with the Student’s t-test (t), was used to compare the average differences between groups or measurements, the usual statistical method to determine the significance of the differences between two sets of data. The confidence interval (95% CI) values ensured a robust and precise estimate of the upper and lower limits of the values, thus consolidating the statistical interpretation. The Pearson correlation coefficient was used to explore the relations between variables, evaluating the power and direction of the linear relationships between the data sets. To protect from Type I error, a Bonferroni correction should be conducted. The new *p*-value will be the alpha value (α_original_ = 0.05) divided by the number of comparisons (2): (α_altered_ = 0.05/2) = 0.025. To determine if either of the 2 correlations is statistically significant, the *p*-value must be *p* < 0.025. The reference for interpretation of Pearson correlations is <0.2 for fairly low, 0.2–0.4 for weak positive relationships, 0.4–0.6 for moderate positive relationships, 0.6–0.8 for fairly strong positive relationships; >0.8 for very strong positive relationships. Linear regression analysis was performed between the variables RAI of RM 4-FM (part 1 and part 2), BMI, and PAI. Cohen’s was calculated to indicate the effect size and was interpreted as follows: <0.3 small effect, >0.3 < 0.5 medium effect, >0.5 > 0.8 large effect, and >0.8 very large effect. With GPower 3.1.9.4. software, we determined the size of the two study groups. The statistical significance threshold is set as *p* < 0.05 in the study, indicating a high level of trust in the results and rejecting the null hypothesis.

## 3. Results

Table 1 provides evidence for the statistical interpretation of the characteristics of the two groups (male and female), and Table 2, Table 3 and Table 4 contain details of the statistical parameters of the two questionnaires applied and the relationships between variables. Results in Table 1 show some important differences between the female and male groups of the study. The male age, 20.417 years old, is higher than that of the female group. The 0.819 difference has statistical relevance due to *p* < 0.01. The residence indicator does not reveal significant differences between the two groups, as most of the subjects reside in urban areas. The BMI indicator shows statistically relevant differences; thus, the male group’s weighted mean is higher than that of the female group by 1.559 kg/m^2^. Cohen’s values highlight a small effect size for age and residence, and a large effect size for BMI.

For the physical activity index (PAI), men show a significantly higher score compared to women (53,482 versus 36,668), with a difference of 16,814 points (*p* < 0.01). This suggests that men engage in more intense or frequent physical activity than women (Table 2). Cohen’s values were > 0.5, the effect size of PAI was large.

Table 3 presents the descriptive statistics for the two parts of the questionnaire RM 4-FM: Motivation for Physical Activity and Motivation for Exercise/Workout. The data are decomposed into specific subscales, and the gender differences are analyzed by test t to compare the means.

Part 1. RM 4-FM: Motivation for Physical Activity (Table 3)

For the External Regulation subscale, the female (X = 2.409, SD = 1.169) group registered lower scores than the male group (X = 2.608, SD = 1.217), which is a statistically relevant difference (t = −2.357, *p* = 0.019). The results show that women had a lower motivation to participate in externally regulated physical activities compared to men. The introjected regulation subscale shows that the difference between the groups is significant (t = −4.609, *p* = 0.000), and men (X = 4.243, SD = 1.488) register higher scores than women (X = 3.774, SD = 1.387), which suggests that men are more motivated by the introjected regulation—that is, by guilt or shame connected to physical activity—than women. For the Identified Regulation subscale, there are no significant differences among the groups. This indicates that both groups (for women, X = 5.242, SD = 1.378, and for men, X = 5.267, SD = 1.097) present similar identified regulated motivation referring to the personal value attributed to the physical activity. For the Intrinsic Motivation subscale, the difference between the male and female groups is not significant (t = −1.718, *p* = 0.086), although men (X = 5.016, SD = 1.229) scored higher than women (X = 4.866, SD = 1.230); this suggests a stronger motivation for physical activity in men. RAI (the relative autonomy index) shows that men (X = 3.775, SD = 3.140) scored lower than women (X = 6.274, SD = 3.738), with a statistically significant difference (t = 10.039, *p* < 0.001). The results indicate women’s stronger motivation and increased autonomy in what physical activity is concerned, while the scores of the male group are lower.

Part 2. RM 4-FM: Motivation for Exercise/Workout (Table 3)

For the External Regulation subscale, the results indicate an insignificant statistical difference (t = 2.065, *p* = 0.039) between the groups, with women (X = 4.624, SD = 1.606) scoring higher than men (X = 4.393, SD = 1.525). This shows that women are influenced more by external regulations concerning physical activity. For the Identified Regulation subscale, the female group (X = 5.113, SD = 1.469) scored higher than males (X = 4.769, SD = 1.478), with a relevant difference (t = 3.280, *p* < 0.001). The result suggests that women are more sensitive to the personal values attributed to physical activities and workouts. The results for the Intrinsic Motivation subscale highlight a relevant difference between the two groups (t = 3.967, *p* < 0.001). Women (X = 5.050, SD = 1.516) scored higher than men (X = 4.617, SD = 1.561), a fact that indicates stronger intrinsic motivation in women compared to men. In the RAI (the relative autonomy index), women (X = 2.336, SD = 4.251) scored insignificantly lower than men (X = 4.624, SD = 1.606), with a relevant difference (t = 2.056, *p* = 0.040). This reveals higher autonomy and positive motivation for workouts in men. Cohen’s values highlight a small effect size for all subscales of Part 1. RM 4-FM: Motivation for physical activity and for Part 2. RM 4-FM: Motivation for exercise/workout, too. The Cohen’s value of RAI of RM 4-FM was > 0.5, indicating a large effect size.

In Table 4, Part 1. RM 4-FM: Motivation for Physical Activity, the correlation among the subscales and the other variables of the research varied between the male and female groups, revealing motivational differences regarding physical activity and workouts. Analyzing the results of the male group for the External Regulation subscale, one can see a moderately positive correlation with the Introjected Regulation, where r = 0.553, *p* < 0.01, Identified Regulation, where r = 0.517, *p* < 0.01, and Intrinsic Motivation, where r = 0.489, *p* < 0.01 subscales. Analyzing the results of the female group for the External Regulation subscale, there is a weak positive correlation with the subscale Introjected Regulation, where r = 0.368, *p* < 0.01, and with Identified Regulation and Intrinsic Motivation, where r < 0.2, *p* < 0.01, the correlations are in the same register. For the male group, the correlations of the Introjected Regulation with the Identified Regulation, where r = 0.383, *p* < 0.01, and Intrinsic Motivation, where r = 0.336, *p* < 0.01, are weakly positive. For the female group, the subscale Introjected Regulation shows a moderately positive correlation with the subscales Identified Regulation, where r = 0.533, and Intrinsic Motivation, where r = 0.511, with *p* < 0.01. The subscale Identified Regulation registered positive correlations with the subscales Intrinsic Motivation r = 0.824 for men and r = 0.829 for women, where *p* < 0.01. The relevant correlations for RAI are moderately negative with the subscale External Regulation: r = −0.388 and *p* < 0.01, and with Introjected Regulation: r = −0.349, *p* < 0.01; moderately positive with Identified Regulation: r = 0.457, *p* < 0.01; and strongly positive with the subscale Intrinsic Motivation: r = 0.564, *p* < 0.01. RAI is inversely proportional with the subscales External Regulation and Introjected Regulation and directly proportional with Identified Regulation and Intrinsic Motivation subscales, indicating a complex relationship. For Age, Residence, and BMI, the correlations between all the subscales and RAI are very weak or irrelevant (*p* > 0.05 for all); these variables do not have statistically relevant connections with the subscales or RAI. PAI registered moderate correlations with the subscales of Introjected Regulation, Identified Regulation, and Intrinsic Motivation with RAI, where r < 0.4 for *p* < 0.01. PAI has positive relationships with the subscales from Part 2, except the External Regulation subscale.

For Part 2, RM 4-FM: Motivation for Exercise/Workout, the results concerning the positive correlations between the variables have partial statistical relevance. Analyzing the results for the subscale Externa Regulation for both groups, we see a moderately positive correlation with the other subscales, with Pearson values between 0.4 and 0.6, *p* < 0.01. Between the subscales External Regulation and RAI, there is a dependently weak negative connection for the female group, −0.345, and a moderate negative relationship for the male group, r = −0.427, *p* < 0.01. The subscale Introjected Regulation had a weak positive correlation with the other subscales, with the Pearson values between 0.2 and 0.4 for the female group and a moderately positive correlation for the male group. There is a weak negative dependency relationship, r < 0.4, between the subscales Identified Regulation and RAI for both of the groups included in the study. It must be noticed that for the subscale Identified Regulation, the correlations with the other subscales are positive for both groups, r = 0.880 for the female and r = 0.882, *p* < 0.01, for the male group. There is a moderately positive correlation between the subscale Identified Regulation and RAI, with r = 0.451 for the female group and r = 0.454 for the male group. Analyzing the Pearson values for the subscales Intrinsic Motivation and RAI, we see a moderately positive relationship, where r = 0.487 for the female group and r = 0.570 for the male group, with *p* < 0.01 (Table 4). Looking at the results of the female and male groups, it can be noticed that the variables age, residence, BMI, and PAI do not correlate with any of the subscales, nor with RAI from Part 2, RM 4-FM: Motivation for Exercise/Workout. The motivation to exercise does not depend on the gender, demographic, and age characteristics of the subjects. Still, the lifestyle or the indicator that highlights the ideal body weight does not matter either.

Linear regression analysis was performed between the variables RAI of each part of RM 4-FM, BMI, and PAI. The regression analysis results demonstrated that the predictor variable, RAI of Part 1. RM 4-FM explained 12% of the variance in BMI for the male group, with statistical significance indicated by [F(1,338) = 1.24, *p* = 0.092]; for the male group, it explained 18% of the variance in BMI for the male group, with statistical significance indicated by [F(1,447) = 3.79, *p* = 0.047]; for RAI of Part 2. RM 4-FM explained 9% of the variance in BMI for the male group, with statistical significance indicated by [F(1,338) = 1.31, *p* = 0.089]; for the male group, it explained 13% of the variance in BMI for the male group, with statistical significance indicated by [F(1,447) = 1.712, *p* = 0.039]. The regression results of the female group showed that the predictor RAI of Part 1. RM 4-FM accounted for 15.7% of the variation in PAI [F(1,447) = 3.57, *p* = 0.040]; for the male group, RAI of Part 1. RM 4-FM accounted for 9.2% of the variation in PAI [F(1,338) = 3.57, *p* = 0.072]. Results of linear regression between RAI of Part 1. RM 4-FM and PAI revealed for the female group R_Square_ = 0.096 (9.6%), [F(1,447) = 1.63, *p* = 0.181]; for the male group R_Square_ = 0.134 (8.1%), [F(1,338) = 1.93, *p* = 0.152].

## 4. Discussion

The results of this study emphasize the complexity of the relationship between motivation, physical activity, and physical health, highlighting significant gender differences and how they influence engagement in physical activities. Intrinsic motivation emerged as a strong predictor of high levels of physical activity, more pronounced among women, which is consistent with previous literature [42,43]. Also, men reported higher PAI but a weaker relationship between intrinsic motivation [36,37] and BMI [12,13], suggesting that the intervention strategies should be personalized. The results indicate significant differences between men and women in the physical activity index (PAI). Thus, men had a significantly higher mean score compared to women, highlighting that male students are more active than female students. This fact may reflect the greater involvement of men in vigorous or more frequent physical activity. While our findings reveal significant correlations between motivation, BMI, and physical activity levels (PAI), it is essential to recognize that the correlation implies weak causality. This study reveals important connections among motivation, body mass index (BMI), and physical activity levels (PAI) in college students, highlighting significant gender differences. The findings show that higher intrinsic motivation is strongly linked to increased physical activity, while the effect of extrinsic motivation differs between genders. However, it is essential to recognize that these relationships are correlational, and the direction and causal mechanisms behind them remain relatively weak [44,45,46,47,48,49]. These insights provide valuable implications for promoting healthier lifestyles among academic students.

Regarding intrinsic motivation [8,9,10], women obtained a slightly higher average score compared to men, this difference being statistically relevant. These results suggest that women are better motivated by the personal satisfaction gained from physical activities, while men are more involved in physical activities, probably due to extrinsic factors [50,51,52,53].

This study’s findings highlight substantial gender differences in physical activity levels (PAI) and motivation. Men generally achieve higher PAI scores, while women exhibit stronger intrinsic motivation. These results are consistent with previous research, which often indicates that men tend to engage in more intense or frequent physical activities, whereas women are largely motivated by intrinsic factors, including personal satisfaction and well-being [54,55]. The study conducted by Sáez I. et al. (2021) on university students’ exercise motivation [56] revealed that men typically score higher on external motivators, such as competition and social recognition. Conversely, women are more influenced by self-determined motivation. Additionally, research by Spiteri et al. (2019) showed that social and environmental factors [57] play a more significant role in motivating men to take part in sports, while women are inclined to pursue physical activity for psychological and health benefits rather than for external rewards. The greater PAI observed in men corresponds with findings from Brazo-Sayavera et al. (2021), which indicated that men are more likely to engage in organized sports and vigorous activities, whereas women prefer low-to-moderate intensity exercise [58].

Motivation plays a critical role in maintaining physical health, and some studies show that psychological factors, such as autonomy and competence, facilitate long-term engagement [25] in physical activities [26,27]. Prior research suggests that extrinsic motivation, although less sustained, could be an initial catalyst for adopting active behaviors. However, the transition to intrinsic motivation is essential for long-term maintenance of benefits [59,60]. In addition, recent research has highlighted the role of interactive technologies, such as exergames [61], in boosting motivation and engagement in physical activities [50]. Not only do these tools improve the appeal of activities but also provide immediate feedback, reinforcing progress and autonomy. The gender differences [51] identified in this study suggest that sports programs should be individualized to address the specific needs of each gender. For example, women may benefit from programs emphasizing social support and personal satisfaction [52], while men may respond better to interventions based on competition and social recognition [53,62]. These findings are supported by studies showing that intrinsic motivation is stronger among women, while men tend to be extrinsically motivated [63,64]. Promoting physical activity through motivational strategies can have a significant impact on public health. Reducing the prevalence of obesity and chronic diseases associated with a sedentary lifestyle requires well-targeted interventions that encourage the adoption of active behavior [65,66]. Studies show that programs based on self-determination theory [8] efficiently promote healthy behaviors because they focus on meeting basic psychological needs [67,68].

The study highlights the need to integrate objective measurements of physical activity with psychological data to provide a holistic perspective on the factors influencing engagement in physical activities [69,70]. In this context, technologies such as activity trackers and mobile applications can facilitate the collection of more accurate and personalized data [71,72]. The statistical analysis showed that men had a higher BMI average compared to women, the difference being statistically relevant, which reflects biological differences and behavioral habits, such as the preference for physical activities that are more important and more frequent in men.

Longitudinal studies could provide additional information on how motivation evolves over the long term and influences health behaviors [73,74]. Additionally, extending the research to other demographic groups could provide a broader perspective on how motivation and physical activity vary by age, cultural context, and socioeconomic status [75,76]. The data obtained revealed a clear relationship between the degree of intrinsic motivation and favorable health outcomes. Women who exhibited higher intrinsic motivation had lower BMI values [77], which supports the hypothesis that personal enjoyment and satisfaction are crucial determinants of engagement in physical activities [78,79,80,81,82]. Although the level of physical activity was higher in men, motivation was influenced more by extrinsic factors such as competition or social recognition [79,83,84,85,86]. This suggests the need for differentiated approaches to foster intrinsic motivation among men. The complex contexts that determine motivation and practice of physical activities must be approached in an interdisciplinary manner to highlight the relevant aspects that determine the formation of proactive behaviors among students with direct effects on physical and mental health [87,88,89,90].

### 4.1. The Limitations of the Study

A significant limitation of this study is its neglect of external variables that may impact the findings. Socioeconomic status, for instance, can affect access to essential resources like nutritious food and physical activity facilities. Psychological factors, such as stress and self-efficacy, also play a crucial role in motivating individuals to exercise. Those with high stress may struggle to maintain a fitness routine, while those with strong self-efficacy are more likely to engage in physical activity. Furthermore, environmental factors, including the availability of sports facilities, are vital to an individual’s ability to lead an active lifestyle. The interactions among these factors may considerably influence motivation and physical activity levels, potentially distorting the observed relationships in this study. This study is limited by its exclusive reliance on self-reported measures of body mass index (BMI), physical activity index (PAI), and RM 4-FM scores, which may introduce potential biases such as recall bias and social desirability effects. Specifically, self-reported BMI does not distinguish between muscle mass and body fat percentage, which can lead to misclassifying individuals with higher muscle mass as overweight. Moreover, self-reported data on physical activity levels may be inaccurate based on personal perceptions. Limitations of the study are the following: the relatively small size of the sample groups may limit the generalization of the results; self-reporting of physical activity levels may introduce reporting bias, especially in participants who underestimate or overestimate the amount of physical exercise; using only two questionnaires (RM 4-FM and PAI) to asses’s motivation and physical activity may not reflect the complexity of factors influencing physical behaviors; limiting the age of subjects included in the study; no motor or functional tests were applied; the lack of intervention programs; the influence of other factors, such as social, eating habits and environmental conditions etc., was not analyzed. A limitation of this study is that while it explores the relationships between motivation, BMI, and PAI, it does not test any intervention programs.

### 4.2. The Practical Implications

The practical implications of the results of this study will focus on the personalization of physical activity programs, gender-adapted; the integration of technologies such as exergames and mobile applications that provide real-time and personalized feedback; promoting health education by implementing workshops on the benefits of physical activity and motivation in the university environment, contributing to the creation of an environment that supports a proactive lifestyle.

## 5. Conclusions

Results revealed significant gender differences: men had a higher PAI than women. This study identified significant gender differences in physical activity levels (PAI), motivation, and BMI among university students. Men had a higher PAI and BMI, while women showed stronger intrinsic motivation for exercise. This study identifies significant associations between motivation, BMI, and physical activity levels (PAI) among college students, with gender differences playing a key role. The relevant results of the study suggest that higher intrinsic motivation may have a more positive impact on physical activity. In contrast, extrinsic forms of motivation may have varying effects by gender. However, these relationships are correlational, and the direction and causal mechanisms behind these associations were relatively weak. The observed discrepancies in physical activity levels (PAI) and motivation between genders indicate that women exhibit higher intrinsic motivation, whereas men participate in higher levels of physical activity. These findings underscore the necessity for gender-specific strategies in the development of interventions aimed at enhancing physical activity participation. The study’s findings suggest the need for educational programs that encourage intrinsic motivation and are adapted for gender, age categories, and social environment. Future research directions will focus on the impact of technology on modeling proactive behaviors to increase fitness in the long term, offering attractive and personalized solutions.

## Figures and Tables

**Table 1:** Descriptive statistics of study group characteristics.

Variable	Group	X	SD	∆X	t	*p*	95% CI		Effect Size
							Lower	Upper	
Age	Female	19.598	1.692	−0.819	−4.074	<0.001	−1.214	−0.424	−0.290
	Male	20.417	3.892						
Residence	Female	1.359	0.480	0.032	0.973	0.331	−0.033	0.099	0.070
	Male	1.326	0.469						
BMI	Female	21.690	3.406	−1.559	−6.207	<0.001	−2.052	−1.066	−0.582
	Male	23.250	3.703						

X—mean, SD—standard deviation, ∆X—difference in means, female–male, t—Student’s t-test value, *p*—probability level, 95% CI—95% confidence interval of the difference.

**Table 2:** Descriptive statistics of the physical activity index (PAI).

Variable	Group	X	SD	∆X	t	*p*	95% CI		Effect Size
							Lower	Upper	
Physical Activity Index (PAI)	Female	36.668	23.922	−16.814	−9.181	<0.001	−20.409	−13.219	−0.796
	Male	53.482	28.114						

X—mean, SD—standard deviation, ∆X—difference in female–male means, t—Student’s t-test value, *p*—probability level, 95% CI—95% confidence interval of the difference.

**Table 3:** Descriptive statistics of the RM4-FM: Motivation for Physical Activity and Exercise/Workout.

Subscale RM 4-FM	Subscale	Group	X	SD	∆X	t	*p*	95% CI		Effect Size
								Lower	Upper	
Part 1. RM 4-FM: Motivation for physical activity	External regulation	Female	2.409	1.169	−0.199	−2.357	0.019	−0.365	−0.033	−0.168
		Male	2.608	1.217						
	Introjected Regulation	Female	3.774	1.387	−0.468	−4.609	<0.001	−0.668	−0.269	−0.328
		Male	4.243	1.488						
	Identified Regulation	Female	5.242	1.378	−0.024	−0.270	0.787	−0.321	0.021	−0.119
		Male	5.267	1.097						
	Intrinsic Motivation	Female	4.866	1.230	−0.150	−1.718	0.086	−0.321	0.021	−0.262
		Male	5.016	1.229						
	RAI	Female	6.274	3.738	2.499	10.039	<0.001	2.010	2.981	0.714
		Male	3.775	3.140						
Part 2. RM 4-FM: Motivation for exercise/workout	External regulation	Female	4.624	1.606	0.231	2.065	0.039	0.011	0.450	−0.108
		Male	4.393	1.525						
	Introjected Regulation	Female	3.651	1.317	0.204	2.184	0.029	0.020	0.388	−0.184
		Male	3.446	1.319						
	Identified Regulation	Female	5.113	1.469	0.343	3.280	<0.001	0.137	0.549	−0.164
		Male	4.769	1.478						
	Intrinsic Motivation	Female	5.050	1.516	0.433	3.967	<0.001	0.218	0.647	−0.101
		Male	4.617	1.561						
	RAI	Female	2.336	4.251	0.601	2.056	0.040	0.0027	1.174	0.572
		Male	4.624	1.606						

X—mean, SD—standard deviation, ∆X—the difference in means, female-male, t—Student’s t-test value, *p*—probability level, 95% CI—95% confidence interval of the difference, RAI—index of autonomy.

**Table 4:** Statistical relevance of Pearson correlations between RM 4-FM: Motivation for Physical Activity, PAI, and group characteristics.

Subscale RM 4-FM		Group	Age	R	BMI	PAI
Part 1. RM 4-FM: Motivation for physical activity	External Regulation	Female	−0.039	0.050	0.043	0.054
		Male	−0.011	0.044	−0.031	−0.024
	Introjected Regulation	Female	0.008	0.013	−0.006	0.235 **
		Male	−0.024	0.020	−0.039	−0.054
	Identified Regulation	Female	0.078	−0.088	−0.059	0.429 **
		Male	−0.045	−0.002	−0.038	−0.042
	Intrinsic Motivation	Female	0.062	−0.098 *	−0.060	0.373 **
		Male	−0.038	0.021	−0.037	−0.068
	RAI	Female	0.094 *	−0.126 **	−0.092 *	0.227 **
		Male	−0.028	−0.022	−0.011	−0.031
Part 2. RM 4-FM: Motivation for exercise/workout	External Regulation	Female	0.050	0.028	0.044	−0.087
		Male	−0.082	0.051	−0.060	0.067
	Introjected Regulation	Female	−0.031	0.009	0.011	−0.082
		Male	0.011	0.023	−0.050	0.008
	Identified Regulation	Female	−0.026	−0.010	0.063	−0.065
		Male	−0.041	−0.003	−0.073	0.042
	Intrinsic Motivation	Female	0.008	0.003	0.056	−0.103
		Male	0.006	0.004	0.045	−0.086
	RAI	Female	0.003	−0.029	−0.003	−0.014
		Male	−0.035	−0.019	0.013	−0.004

**. Correlation is significant at level 0.01 (2-tailed); *. Correlation is significant at level 0.05 (2-tailed). R—medium of residence, RAI—relative autonomy index, BMI—body mass index, PAI—physical activity index.

## Data Availability

The original contributions are included in the article.
